# Rapid UHPLC-MS metabolite profiling and phenotypic assays reveal genotypic impacts of nitrogen supplementation in oats

**DOI:** 10.1007/s11306-019-1501-x

**Published:** 2019-03-12

**Authors:** J. William Allwood, Yun Xu, Pilar Martinez-Martin, Raphaёlle Palau, Alexander Cowan, Royston Goodacre, Athole Marshall, Derek Stewart, Catherine Howarth

**Affiliations:** 1 Environmental and Biochemical Sciences, James Hutton Institute, Invergowrie, Dundee, DD2 5DA Scotland, UK; 2 Manchester Institute of Biotechnology, School of Chemistry, University of Manchester, Princess Street, Manchester, M1 7DN UK; 3 Department of Biochemistry, Institute of Integrative Biology, University of Liverpool, Biosciences Building, Crown Street, Liverpool, L69 7ZB UK; 4 Institute of Biological, Environmental & Rural Sciences (IBERS), Aberystwyth University, Plas Gogerddan, Aberystwyth, Ceredigion, SY23 3EE UK; 5 School of Engineering and Physical Sciences, Institute of Mechanical, Process and Energy Engineering, Heriot-Watt University, Edinburgh, EH14 4AS Scotland, UK

**Keywords:** Metabolomics, UHPLC-PDA-MS, Oats, *Avena sativa* L., Nitrogen, Grain quality, β-Glucan, Amino acids, Lipids, Avenanthramides

## Abstract

**Introduction:**

Oats (*Avena sativa* L.) are a whole grain cereal recognised for their health benefits and which are cultivated largely in temperate regions providing both a source of food for humans and animals, as well as being used in cosmetics and as a potential treatment for a number of diseases. Oats are known as being a cereal source high in dietary fibre (e.g. β-glucans), as well as being high in antioxidants, minerals and vitamins. Recently, oats have been gaining increased global attention due to their large number of beneficial health effects. Consumption of oats has been proven to lower blood LDL cholesterol levels and blood pressure, thus reducing the risk of heart disease, as well as reducing blood-sugar and insulin levels.

**Objectives:**

Oats are seen as a low input cereal. Current agricultural guidelines on nitrogen application are believed to be suboptimal and only consider the effect of nitrogen on grain yield. It is important to understand the role of both variety and of crop management in determining nutritional quality of oats. In this study the response of yield, grain quality and grain metabolites to increasing nitrogen application to levels greater than current guidelines were investigated.

**Methods:**

Four winter oat varieties (Mascani, Tardis, Balado and Gerald) were grown in a replicated nitrogen response trial consisting of a no added nitrogen control and four added nitrogen treatments between 50 and 200 kg N ha^−1^ in a randomised split-plot design. Grain yield, milling quality traits, β-glucan, total protein and oil content were assessed. The de-hulled oats (groats) were also subjected to a rapid Ultra High Performance Liquid Chromatography—Mass Spectrometry (UHPLC-MS) metabolomic screening approach.

**Results:**

Application of nitrogen had a significant effect on grain yield but there was no significant difference between the response of the four varieties. Grain quality traits however displayed significant differences both between varieties and nitrogen application level. β-glucan content significantly increased with nitrogen application. The UHPLC-MS approach has provided a rapid, sub 15 min per sample, metabolite profiling method that is repeatable and appropriate for the screening of large numbers of cereal samples. The method captured a wide range of compounds, inclusive of primary metabolites such as the amino acids, organic acids, vitamins and lipids, as well as a number of key secondary metabolites, including the avenanthramides, caffeic acid, and sinapic acid and its derivatives and was able to identify distinct metabolic phenotypes for the varieties studied. Amino acid metabolism was massively upregulated by nitrogen supplementation as were total protein levels, whilst the levels of organic acids were decreased, likely due to them acting as a carbon skeleton source. Several TCA cycle intermediates were also impacted, potentially indicating increased TCA cycle turn over, thus providing the plant with a source of energy and reductant power to aid elevated nitrogen assimilation. Elevated nitrogen availability was also directed towards the increased production of nitrogen containing phospholipids. A number of both positive and negative impacts on the metabolism of phenolic compounds that have influence upon the health beneficial value of oats and their products were also observed.

**Conclusions:**

Although the developed method has broad applicability as a rapid screening method or a rapid metabolite profiling method and in this study has provided valuable metabolic insights, it still must be considered that much greater confidence in metabolite identification, as well as quantitative precision, will be gained by the application of higher resolution chromatography methods, although at a large expense to sample throughput. Follow up studies will apply higher resolution GC (gas chromatography) and LC (reversed phase and HILIC) approaches, oats will be also analysed from across multiple growth locations and growth seasons, effectively providing a cross validation for the results obtained within this preliminary study. It will also be fascinating to perform more controlled experiments with sampling of green tissues, as well as oat grains, throughout the plants and grains development, to reveal greater insight of carbon and nitrogen metabolism balance, as well as resource partitioning into lipid and secondary metabolism.

**Electronic supplementary material:**

The online version of this article (10.1007/s11306-019-1501-x) contains supplementary material, which is available to authorized users.

## Introduction

Oats, *Avena sativa* L., are a whole grain cereal recognised for their health benefits and which are cultivated largely across Europe, Russia and North America, providing both a source of food for human consumption and more significantly for animal-feeds, as well as being used in cosmetics as a source of emollients (Marshall et al. 2013; Heuzé et al. 2016) and applied to the treatment of dysmenorrhoea, osteoporosis and urinary tract infection, amongst other diseases (Duke, 2002). In 2016 global oat production was 23 million tonnes, with the largest producers being Russia (23%) and Canada (13%) (FAO 2017). Oats are thought to have originated from the Fertile Crescent within the modern-day middle east and throughout their domestication were spread into western Europe (Zhou et al. 1999). Compared to the other major cereal species, oats have much lower summer heat requirements and much greater tolerance of rain, and thus are very well suited to cultivation in conditions such as those found across North Western Europe and North America. Oats are known as being a cereal source high in dietary fibre (e.g. β-glucans), with the oat itself containing anywhere up to 11% total fibre content, as well as being high in antioxidants (ferulic acid, phytic acid, avenanthramides), minerals and vitamins (manganese, copper, phosphorus, iron, selenium, magnesium, zinc, B Vitamins—Thiamine, Riboflavin, Niacin, Pantothenic acid, B6, and Vitamin E—tocols) (Peterson 2001; Rasane et al. 2015). Oats are also recognised as being well nutritionally balanced, especially within the oat bran with respect to carbohydrate (66%), protein (17%) and fat (7%) content (USDA, National Nutrient Database for Standard Reference Legacy Release, Oat Bran Raw—Report No. 20033).

Recently, oats have been gaining increased global attention due to the recognition of not just their nutritional balance, but due to their large number of beneficial health effects. Oats are low in free sugars, but high in fibre, the majority of which (85%) are starches. Oat starch and β-glucan based fibre are much more abundant and also more readily digested than fibre in the alternative major cereal grains, which results in a slower digestive transit, increased satiety and appetite suppression (Clark and Slavin 2013). Oats also contain a large proportion of non-digestible starch, 25% of total levels, these non-digestible starches are thought to improve gut health by providing a source of nutrition to gut-beneficial bacteria (Nugent 2005). Research has repeatedly illustrated that oats, especially when consumed as oat bran or oatmeal, do lower blood LDL cholesterol levels, thus reducing the risk of heart disease (Andon and Anderson 2008; Berg et al. 2003; Bernstein et al. 2013; Tighe et al. 2010; Truswell 2002; Whitehead et al. 2014). In fact, this soluble fibre-derived effect has led to the producers of oat products making health claims regarding a reduced risk of cardiovascular disease and cholesterol reduction. To qualify such claims, the administrative authorities in the USA and EU have stated that “the whole oat-containing food must provide at least 0.75 g of soluble fibre per serving. The amount of soluble fibre needed for an effect on cholesterol levels is about 3 g per day. Soluble fibre must be β-glucan from oat bran, rolled oats or oatmeal, or oat flour. Oat bran must contain 5.5% β-glucan and oatmeal and oat flour must contain 4% β-glucan” (Stewart et al. 2014).

Studies have also shown that a diet rich in oat bran and oatmeal can directly be correlated with reduced blood pressure levels (He et al. 2004; Keenan et al. 2002), as well as reducing blood-sugar and insulin levels (Alminger and Eklund-Jonnson 2008; Tosh 2013), the improved control of which can aid a reduction in obesity and Type II diabetes prevalence (Cho et al. 2013; Ye et al. 2012). Oats are a particularly rich source of anti-oxidants, including high levels of both ferulic and phytic acids, as well as a group of anti-oxidants that are unique to oats and which are known as the avenanthramides. Avenanthramides are believed to contribute to reductions in arterial inflammation as well as having positive effects on the regulation of blood pressure (Liu et al. 2004; Meydani 2009; Nie et al. 2006). It is also believed that storage proteins in oats known as avenins (gliadins in wheat, hordeins in barley, secalins in rye), are less liable to cause toxicity to sufferers of celiac disease, although it is thought to be dependent on oat variety as well as considerations that oats must be processed in an environment clean of other cereals. Therefore, oats have been proposed as being safe to consume as part of a gluten free diet, although it remains controversial since very low numbers of sufferers of celiac disease still react to oat avenins (Comino et al. 2015; de Souza et al. 2016).

In the United Kingdom, winter oats are sown in late September/early October and are harvested the following August. Compared to other cereals (e.g. wheat and barley), oats are seen as a low input cereal needing lower fertilization levels (Weightman et al. 2004; Kindred et al. 2008). The effects of nitrogen application during oat yield development on grain quality parameters are however poorly understood compared with wheat and barley. Provision of a sufficient amount of nitrogen, usually in the form of urea or anhydrous ammonia, has great bearing on the plant height, quality and oat yield (Chalmers et al. 1998; Givens et al. 2004; Yan et al. 2017). Oat quality and yield in turn impact upon the processing quality of oats, with respect to dehulling, as well as the later processing steps of flaking and milling (Browne et al. 2003). Kernel content (the proportion of groat to whole grain) and ease of hull removal (hullability) are the most important traits for milling quality (Browne et al. 2003). Current agricultural guidelines on the levels of nitrogen application in the UK. do not currently consider the effect on milling quality or grain composition. It is important also to understand the role of variety choice and of crop management in determining nutritional quality of oats. To study this further, four winter oat varieties were grown in a replicated nitrogen response trial. The trial consisted of a no added nitrogen control and four added nitrogen treatments. Grain yield, yield components and milling quality traits were assessed. The de-hulled oats (groats) were also subjected to a rapid Ultra High Performance Liquid Chromatography - Mass Spectrometry (UHPLC-MS) metabolomic screening approach (Allwood and Goodacre 2010; Allwood et al. 2011; Koistinen and Hanhineva 2017), revealing the impact of nitrogen supplementation on a range of compounds inclusive of amino acids, organic acids, lipids and phenolics.

## Materials and methods

### Field trial design

The trial was conducted at Lydbury North, Shropshire, UK (latitude 52.45, longitude − 2.94, medium soil type) in a split plot design with three replicates using five levels of nitrogen application (main plot treatment) and four commercially available winter oat varieties (sub-plot treatment) from the Aberystwyth University winter oat breeding programme. These included two of the most widely grown winter oat varieties in the UK over the last 20 years, Gerald and Mascani (AHDB 2015). Three varieties were of conventional height (Mascani, Gerald and Tardis) and the fourth was a dwarf variety (Balado). Plots (1.8 × 6 m) were sown on the 9th October 2013 at a sowing rate of 300 seeds m^−2^ and harvested on the 7th August 2014 using a small plot combine. Fungicides and weed control followed standard practise for winter oats including the use of a plant growth regulator (Cycocel 5C). Soils were sampled to a depth of 90 cm in early spring and residual soil nitrogen was determined to be 58 kg N ha^−1^. Nitrogen (ammonium nitrate) doses were split between three different developmental stages in early to late spring as indicated in Table S1 to provide five final doses: Control, no applied nitrogen; level 1, 50 kg N ha^−1^ applied; level 2, 100 kg N ha^−1^ applied; level 3, 150 kg N ha^−1^ applied; level 4, 200 kg N ha^−1^ applied. The 17th April application date corresponded to growth stage 31 (early stem elongation, Zadoks et al. 1974). Prior to harvest the number of fertile shoots (panicles) per m^2^ were counted and plant heights from soil surface to panicle tip measured.

### Chemicals

Unless otherwise stated all solvents were of HPLC grade and JT Baker brand (Scientific Chemical Supplies, UK), formic acid was of MS grade (Fisher Scientific, UK), morin-hydrate and reserpine (99% purity) were obtained from Sigma-Aldrich UK, all other reference standards, unless otherwise stated, were obtained from LGC (UK) or Extrasynthese (FR).

### Grain quality assays

Harvested grain was cleaned through a 3.5 mm and 2 mm sieve to remove broken grain and residual chaff and straw prior to analysis of grain quality. Groats were obtained by passing 25 g of whole grain through a Laboratory Oat Huller (Codema Model LH5095; Maple Grove, Minneapolis, USA) set at 100 bar for 60 s and then separating the output into groats, husks and any un-dehulled whole grain remaining. Kernel content was calculated as the weight of the groats obtained as a proportion of the initial grain weight less the weight of un-dehulled whole grain remaining at the end of the dehulling procedure. Thousand grain weight (TGW) of grain prior to dehulling and of groats was determined using a seed counter (Data technologies model number data count S-25). Grain numbers per m^2^ and per panicle were calculated using grain yield and TGW results.

Nitrogen and oil content of groats were predicted using near infrared spectroscopy (NIRS). Approximately 20 g of cleaned, dehulled groats were analysed by scanning in a transport quarter cup at 2 nm intervals over the wavelength range from 400 to 2498 nm in reflectance mode, using a NIRSystems 6500 spectrophotometer (FOSS UK, Warrington, UK). Data were collected using ISI software (Infrasoft International, Port Matilda, USA) and spectra were stored as log(1/R) where R is the diffuse reflectance. The calibration equations used were developed for oil and nitrogen content in groat samples, ground through a 1 mm sieve and originating from multiple harvest years and trial sites and including both spring and winter varieties, were used for prediction. Nitrogen calibration data was obtained by a rapid combustion method using a LECO FP-428 analyser (LECO Corp., St. Joseph, MI). Oil calibration data was obtained by extraction using petroleum ether and the Soxtec system (FOSS UK, Warrington, UK). Calibration equations were developed using standard normal variate and detrend (Barnes et al. 1989) and second derivative transformations using modified partial least squares regression. β-glucan content was determined on a subsample of ground groat using the Megazyme™ kit (McCleary method) (AOAC method 995.16, 1999) on all samples. The data was analysed using 2-way analysis of variance and Fisher’s protected least significance test in GENSTAT 16th edition and correlation analysis conducted using Microsoft Excel.

### UHPLC-PDA-MS extraction

The dehulled oat samples were first homogenised using a Retsch Cyclone Mill—Twister with the following settings: speed 12,000 rpm min^−1^; 62 milli second peripheral rotor speed; 1 mm sieve; 1 min cycle. 100 mg ± 2 mg FW of oats were weighed into 2 mL safe-lock Eppendorf microcentrifuge tubes, 1.5 mL of extraction buffer (10 uM reserpine and 10 uM morin in 75% methanol 24.9% water 0.1% formic acid) was added, the sample was vortexed for 15 s, ultrasonicated for 15 min with an Elma S40 ultrasonicator, vortexed for 15 s, agitated on an IKA VXR basic vibrax fitted with the VX2E.n Eppendorf tube adapter set on speed 8 for 30 min, and centrifuged for 10 min at 3 °C and 18,407×*g* with an Eppendorf 5424R (rotor FA-45-24-11). The extract supernatants were next filtered with 0.45um PTFE filter vials (Thomson single step) and transferred to 2 mL HPLC vials with pre-slit caps (Thermo-Fisher, Chromacol 2SVW and 9-SCK(B)-ST1 X, respectively). A quality assurance (QA) sample was prepared by mixing equal quantities of each sample extract, thus providing a QA sample representative of the average of the entire sample set. A number of representative samples were also extracted and analysed in triplicate (denoted as a, b, and c, within the sample number) to serve as reference samples to define extraction and analytical repeatability. The samples were stored in the HPLC autosampler at 10 °C and analysed within 72 h of extraction in positive and negative electrospray ionisation (ESI) modes. The ESI source spray cone and ion tube were cleaned between running the sample set in each ionisation mode.

### UHPLC-PDA-MS analysis

UHPLC separations were performed with a Thermo Accela 600 HPLC system coupled with an Accela PDA detector (Thermo-Fisher Ltd. U.K). The HPLC was operated at a flow rate of 400 µL/min, the column and guard column (Thermo Hypersil Gold C18 50 × 2.1 mm, 1.9 µm particle size; Hypersil Gold C18 Guard 10 × 2.1 mm, 3 µm particle size; Thermo Universal Uniguard guard holder 2.1/3 mm) were maintained at a temperature of 40 °C. The solvent A, HPLC grade water, and solvent B, HPLC grade acetonitrile, were acidified with 0.1% [v/v] MS grade formic acid. Prior to sample analysis a new UHPLC column and guard column were conditioned with solvents A and B for a minimum of 40 min at a flow rate of 400 µL/min. A sample injection volume of 5 µL was employed in full-loop mode. The gradient programme was as follows: hold 5% B 0–1 min, 5–100% B 1–8 min, hold 100% B 8–11 min, 100–5% B 11–11.1 min, hold 5% B 11.1–15 min. Autosampler syringe and line washes were performed with 80% HPLC grade acetonitrile 20% HPLC grade water. The UHPLC column eluent was first monitored by the Accela PDA detector where spectra were collected in wavelength/absorbance mode from 200 to 600 nm with a filter bandwidth and wavelength step of 1 nm, the filter rise time was 1 s, the sample rate was 10 Hz. Additionally three channel set points were employed, Channel A 280 nm, Channel B 365 nm, Channel C 520 nm, with a bandwidth of 9 nm and a sample rate of 10 Hz.

The PDA detector eluent was next transfered to a Thermo LTQ-Orbitrap XL mass spectrometry system operated under Xcalibur software (Thermo-Fisher Ltd. UK). Mass spectra were primarilly collected in full scan mode (*m*/*z* 100–1200) at a mass resolution of 30,000 (FWHM defined at *m*/*z* 400) within the FT detector for all samples. An additional method was applied to obtain ion trees by performing data-dependent analysis (DDA) MS2 on the top 3 most intense ions for the mixed QA sample (Mullard et al. 2015). The DDA method applied a primary full scan event within the FT, followed by a secondary scan event within the LTQ-IT to collect MS^2^ CID fragmentation spectra for the top 3 most intense ions as defined within the preliminary full MS scan. Helium was applied as a collision gas for CID at a normalised collision energy of 45%, a trapping window width of 2 (± 1) *m*/*z* was applied, an activation time of 30 ms and activation Q of 0.25 were applied, only singly charged ions were selected for DDA, isotopic ions were also excluded. The preliminary full scan event within the FT generated ‘profile’ mode data, whereas the LTQ-IT MS2 data were collected in ‘centroid’ mode.

A scan speed of 0.1 s and 0.4 s were applied in the LTQ-IT and FT-MS respectively. The Automatic Gain Control was set to 1 × 10^5^ and 1 × 10^6^ for the LTQ-IT and FT-MS respectively. Prior to the analytical run the LTQ-IT and FT-MS were tuned to optimise conditions for the detection of ions in the mid detection range of *m*/*z* 100–1200, as well as being calibrated with the manufacturers recommended calibration mixture and procedures. The ESI conditions were optimised to allow efficient ionisation and ion transmission whilst limiting insource fragmentation. The following settings were applied to ESI: Spray voltage − 3.5 kV (ESI−) and + 4.5 kV (ESI+); Sheath gas 60; Auxiliary gas 30; Capillary voltage − 35 V (ESI−) + 35 V (ESI+); Tube lens voltage − 100 V (ESI−) and + 100 V (ESI+); Capillary temperature 280 °C; ESI probe temperature 100 °C.

The samples were analysed in a completely randomised order as two independent analytical blocks, each of approximately 100 injections, respective of ESI positive and ESI negative polarities. For each analytical block, initially eight injections of QA sample were performed for LC-MS system conditioning, after which three further injections of QA sample were performed, followed by six injections of experimental samples and a further QA injection. This was repeated until all samples were analysed, finally the analytical block was concluded with a further three QA injections. A control blank sample was analysed at the start and end of the analytical block, thus providing a measure of the sample back-ground and also a measure of compound carry over resulting from dirtying of the ESI source. Each analytical block was finally concluded by collection of the DDA MS2 profiles for the QA sample.

### UHPLC-PDA-MS data processing and peak annotation

The UHPLC-PDA-MS raw data profiles were first converted into an MZML centroid format within the Proteowizard (http://proteowizard.sourceforge.net/) MSConvert software package. Each MZML based three-dimensional data matrix (intensity × *m*/*z* × time − one per sample) was converted (or deconvolved) into a vector of *peak responses*, where a *peak response* is defined as the sum of intensities over a window of specified mass and time range (e.g. *m*/*z* = 102.1 ± 0.01 and time = 130 ± 10 s). In this experiment the deconvolution was performed using the freely available XCMS online package (https://xcmsonline.scripps.edu/). XCMS online was operated with the following parameter set points: Feature detection; method—CentWave; mass error 5 ppm, minimum and maximum peak width 5 and 30 s respectively, mzdiff 0.01, S/N threshold 10, integration method 1, prefilter peaks 3, prefilter intensity 40,000, noise filter 100,000: RT correction; method – Obiwarp, profstep 1: Alignment; minfrac 0.5, mz width 0.015, bw 5, min samp 1, max samp 100: Annotation; Search for isotopes + adducts, mz absolute error 0.015, ppm error 5.

The XCMS deconvolution results in the production of a Microsoft Excel based XY matrix containing the paired RT and *m*/*z* of each feature, along with the peak area in each profiled sample, and where provided, adduct and isotope annotations for each *m*/*z*. The XY matrix was trimmed of peaks that eluted within the first 0.3 min and final 4 min of chromatography (the void and equilibration periods), as well as removal of peaks that were dominant within blank sample extracts (more than 2 × more intense than the peaks highest intensity within a biological sample) (Di Guida et al. 2016). Applying a set of workflows known as PutMedID which are operated within the Taverna Workbench 1.7.2 software package (Brown et al. 2009, 2011; Allwood et al. 2013), peak to peak Pearson correlations were first calculated within a ± 5 s moving RT window, peaks that show a high level of Pearson correlation (greater than 0.8) within the same RT window were grouped as *m*/*z* features that were likely associated with the same compound (i.e. an *m*/*z* group). As a second step, accurate mass differences between *m*/*z* within each peak group were calculated to allow the annotation of the parent *m*/*z* from isotopic and adduct ions. Consensus was drawn between the two methods of peak annotation performed within XCMS online and PutMedID. Once the parent ion, adducts and isotopes are annotated within each peak group, the neutral accurate mass is calculated for each ion (5 ppm mass error) and in turn matched to possible molecular formula(s), which are then matched to metabolite name(s). Molecular formula and metabolite matching were based upon a library of known plant metabolites obtained from the Plant Metabolic Network PlantCyc database (http://www.plantcyc.org) in addition to the Manchester Metabolomics Database (MMD: http://dbkgroup.org/MMD/), thus in most cases providing an MSI level 2 or 3 identification (Sumner et al. 2007). Where multiple putative molecular formula or metabolite identifications were obtained by the peak annotation approach, the identifications were also manually checked for having been previously reported as an oat metabolite (FooDB: http://foodb.ca/), thus increasing confidence in the assigned putative identification(s). In the case of lipids where multiple isomeric species could be assigned to a given accurate mass, the lipids fatty acid chain lengths and numbers of unsaturated bonds were summed in order to condense the high numbers of isomeric identifications.

### Chemometric analysis of UHPLC-PDA-MS metabolite profiles

Prior to statistical analysis, the peak area data were normalised to the internal standard peak area, reserpine [M+H]^+^ and morin [M−H]^−^ in positive and negative mode ESI, respectively. Missing values were automatically replaced with a value representative of one-third of the minimum peak ratio across the entire data matrix (Di Guida et al. 2016). The relative standard deviation for each detected feature was calculated across the QA samples, features showing greater than a 25% RSD within QA samples were excluded from statistical tests. Principal Components Analysis (PCA) was performed with the SIMCA-P + 12.01 64bit statistics package (Umetrics, Umeå, Sweden). Two PCA-X models were generated, the first for the dataset inclusive of QA samples, the second for the dataset after exclusion of QA samples. PCA scores plots were generated for PC1 × PC2, PC1 × PC3, PC1 × PC4, PC1 × PC5, PC2 × PC3, PC2 × PC4, PC2 × PC5, PC3 × PC4, PC3 × PC5 and PC4 × PC5. Complementary PCA loadings plots were also generated for the same PC combinations. In addition to PCA, ANOVA-simultaneous component analysis (ASCA) was applied as a method of variable selection (Jansen et al. 2005a, b). The constructed ASCA model was designed to test two factors, (i) oat variety, (ii) nitrogen level, with the significance level of the effect of each factor being evaluated by performing 1000 permutation tests (Zwanenburg et al. 2011). The ASCA based results were visualised as PCA score and loading plots. Partial Least Squares with structured output (PLS-S) was employed as a supervised machine learning model (Xu et al. 2016). Unlike conventional PLS regression (PLS-R) or PLS for discriminant analysis (PLS-DA), PLS-S employed a structured response matrix ***Y*** which was coded according to the experimental design and was able to investigate all studied factors simultaneously (e.g. oat varieties and nitrogen levels in this study). Recursive feature elimination (RFE; Guyon and Elisseef 2003) was applied with the PLS-S to identify the metabolites that changed significantly in response to increasing nitrogen level regardless of the oat variety. The PLS-S models with RFE were validated with 1000 bootstrapping re-sampling (Efron and Tibshirani 1994). Finally, a non-parametric univariate significance test based upon two-way ANOVA, i.e. the Friedman test, was performed, a False Discovery Rate (FDR) correction of 5% was applied based upon the Benjamini-Hochberg procedure. Univariate comparisons were made between oat samples of each individual variety separately, to define metabolite features that responded in a linear fashion with respect to increasing nitrogen application level. Univariate tests were performed within MATLAB 2016a using the statistics and machine learning toolbox (Mathworks, MA, US). ASCA and PLS-S with RFE were performed within MATLAB 2016a using in-house scripts which are made available freely online at https://github.com/Biospec/cluster-toolbox-v2.0.

## Results and discussion

### Oat yield and grain quality

Application of nitrogen had a significant effect on grain yield but there was no significant difference in the response of the four varieties (Table 1). Grain yields at the highest nitrogen level (200 kg N ha^−1^) were 130% higher than that without nitrogen fertiliser. It was not possible to determine a nitrogen optimum for grain yield as yields were still increasing substantially up to the highest nitrogen level used.

**Table 1:** Phenotypic analyses

	Plant height mm	Grain yield t ha^−1^	Panicle number m^−2^	Grain number panicle^−1^	Grain number m^−2^	Kernel content %	TGW grain g	TGW groat g	Protein % DM	Oil % DM	β-glucan % DM
Variety											
Gerald	112.80b	8.70	392.00bc	48.21c	18942.62c	73.90b	38.94a	29.59a	10.70	7.66b	3.31a
Mascani	114.00b	8.29	399.78c	37.98a	15307.64a	77.23c	45.85d	37.33d	10.71	7.31a	3.96c
Tardis	112.27b	8.72	363.22b	47.82b	17563.82b	72.64a	42.57b	32.18b	11.01	8.19c	3.75b
Balado	99.00a	8.86	300.22a	56.64d	17128.92b	72.22a	43.76c	35.14c	10.82	7.68b	4.50d
Nitrogen application (kg N ha^−1^)											
0	83.80a	4.90a	251.80a	40.40a	10097.00a	72.98a	41.49a	32.47a	9.11a	8.00e	3.72a
50	101.10b	8.05b	353.70b	44.73b	15603.00b	73.47ab	44.11c	33.46b	9.64b	7.89d	3.83ab
100	111.90c	9.26c	414.60c	45.98bc	18427.00c	73.94bc	42.96bc	33.55b	10.67c	7.70c	3.86ab
150	126.40d	9.74c	408.10c	49.17c	19772.00d	74.10c	42.03ab	33.66b	11.65d	7.57b	3.93bc
200	124.30d	11.26d	390.80c	58.03d	22280.00e	75.50d	43.32c	34.65c	12.97e	7.39a	4.07c
N treatment p value	< 0.001	< 0.001	< 0.001	< 0.001	< 0.001	< 0.001	< 0.001	0.002	< 0.001	< 0.001	0.002
Variety (V) p value	< 0.001	0.164	< 0.001	< 0.001	< 0.001	< 0.001	< 0.001	< 0.001	0.277	< 0.001	< 0.001
V × N p value	0.329	0.290	0.506	0.114	0.101	0.011	0.015	0.023	0.760	0.222	0.563

Mean values by variety and by nitrogen application for plant height, grain yield (at 15% moisture content) and component traits, thousand grain weight (TGW) of grain and de-hulled groat and macro nutrient composition expressed as % dry matter (DM). Replication *n*3. Different letters indicate significant differences between mean values at p < 0.05 as calculated by Fisher’s least significance difference test. N treatment and Variety *p* values (and their interaction) and the level of significance of these factors as calculated by two-way ANOVA analysis

Grain yield was highly correlated with the number of grains m^−2^ (r = 0.99). Grain number m^−2^ is the product of panicle number m^−2^ and grain number per panicle both of which significantly increased with increasing nitrogen treatment. This suggests that greater nitrogen availability resulted in an increased productive tiller survival as previously found (Weightman et al. 2004; Browne et al. 2006; Ma et al. 2017). Panicle number m^−2^ however did not continue to increase after applications of 100 kg N ha^−1^. Grain number per panicle was highest at 200 kg N ha^−1^. A significant difference was found for the yield component traits grain number m^−2^, grain number per panicle and panicles m^−2^ between varieties. Mascani had the lowest grain number per panicle and the highest panicle number m^−2^ (Table 1). Balado and Gerald had the highest grain number per panicle. Competition amongst a greater number of grains can lead to incomplete grain filling resulting in small grains (Marshall et al. 2013). The results suggest that the different varieties studied can adjust their yield component structure depending on the level of nitrogen application but that the actual grain yield was not different between the varieties.

Grain yield was not correlated with grain TGW (*r* = 0.08). Grain TGW was however significantly different both between varieties and nitrogen levels but there was no relationship between nitrogen level and TGW. Groat TGW did increase significantly in response to nitrogen application. There was a significant interaction between nitrogen and variety for TGW. Mascani and Balado both responded to increased nitrogen application by increasing grain and groat size whereas the TGW of Tardis and Gerald remained similar at all nitrogen levels (Table 1; Table S2). A number of studies have reported that the higher the amount of nitrogen applied to the crop the lower the individual grain weight (Peltonen-Sainio and Peltonen 1995; Chalmers et al. 1998; Weightman et al. 2004; Ma et al. 2017). The study reported here indicates that the response of TGW to nitrogen is dependent on the variety under study. TGW is often used as an indicator of grain quality as it is related to grain plumpness and a high value reflects a well-filled grain. Mascani had the highest groat and grain TGW at all nitrogen application levels (Tables 1, S2).

The milling quality trait, kernel content displayed significant differences between varieties and nitrogen application with the highest values obtained by Mascani and at 200 kg N ha^−1^. The difference between varieties was greater than the difference between nitrogen treatments. A similar result was found by Yan et al. (2017). Plant height also increased with nitrogen application (Table 1) as found in other studies (May et al. 2004; Ma et al. 2017) indicating that the increased yield and grain quality at higher N levels could come with an associated higher risk of lodging (Tumino et al. 2017).

Total protein content as measured by NIR significantly increased with nitrogen application but there was no significant difference between the four varieties (Table 1). Total oil content however was significantly different between varieties and decreased significantly in response to nitrogen as also found by Yan et al. (2017). Mascani had the lowest total oil content. β-glucan content significantly increased with nitrogen application with Balado having the highest mean value. Previous studies reporting the effects of nitrogen application on β-glucan content have given conflicting results (Saastamoinen et al. 1992; Brunner and Freed 1994; May et al. 2004; Stewart and McDougall 2014; Yan et al. 2017) suggesting that there is a large genotypic influence on this trait.

The raw data for all phenotypic assays is available within Table S2.

### Rapid UHPLC-PDA-MS of oat grains provides robust metabolite profiles that are information rich and representative of a wide range of metabolite classes

**Fig. 1:**
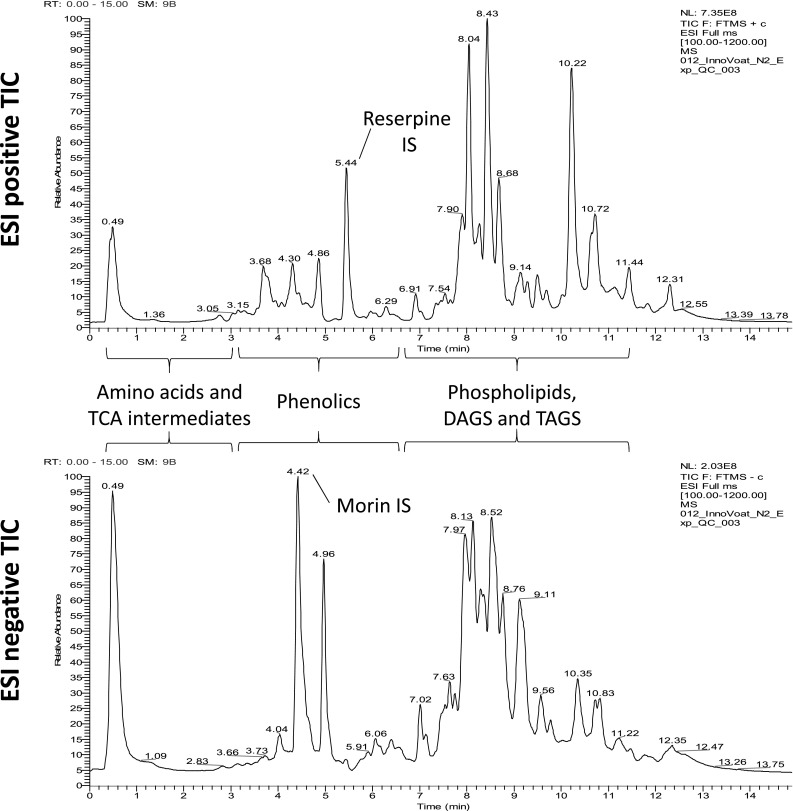
LC-MS oat extract total ion chromatograms (TIC)

UHPLC-PDA-MS profiling of oats produces rich metabolite profiles representative of an impressive range of metabolite classes, inclusive of primary metabolites such as amino acids and TCA intermediates, as well as secondary metabolites such as lipids, vitamins and a number of phenolics such as the avenanthramides (Fig. 1). The deconvolution of these UHPLC-PDA-MS profiles within XCMS online, results in the generation of highly information rich datasets. After filtering (blanks, void and equilibration peaks), the raw integrated peak areas for each RT-*m*/*z* pair were next normalised to the internal standard, thus providing a normalised peak response ratio. Finally, the peaks were filtered that failed a 25% RSD cut off across the QA samples. Following filtering and QA, the ESI positive mode dataset contained a total of 3214 deconvolved RT-*m*/*z* pairs, and the ESI negative mode dataset a total of 1558.

Post filtering and QA, Pearson correlations were calculated to group *m*/*z* features that were likely associated with the same compound (i.e. an *m*/*z* group). Within each *m*/*z* group the peaks were annotated as parent, isotope and adduct ions, as well as common in-source fragments, the neutral accurate mass was calculated and in turn matched to a library of possible molecular formulae and associated metabolite names. Post annotation, all grouped RT-*m*/*z* pairs, whether a compound annotation had been achieved or not, were taken forward. Finally, within each dataset, the *m*/*z* groups were further filtered to remove redundant isotope and adduct features, thus further assisting in reducing data complexity and aiding downstream interpretation. The filtered ESI positive and negative mode datasets contained 2012 (Table S3) and 924 RT-*m*/*z* pairs (Table S4), respectively, that were taken forward to statistical analysis. Of the total 2012 ESI positive and 924 ESI negative RT-*m*/*z* pairs, 914 ESI positive and 480 ESI negative were identified to MSI level 2 or level 3 by matching of HRMS accurate mass data, and 1093 ESI positive and 436 ESI negative were classed as MSI level 4 unknowns (Sumner et al. 2007). Within Tables S3 and S4 the MSI identification levels and where applicable PubChem CID identifiers are provided. All possible identifications for each RT-*m*/*z* pair are provided in Table S3 and S4, whereas just the most likely identification(s) based upon compounds previously reported within oats (FooDB: http://www.foodb.ca), or more widely the plant kingdom, are provided in Tables S5 and S6.

### Chemometric analysis of UHPLC-PDA-MS oat profiles

**Fig. 2:**
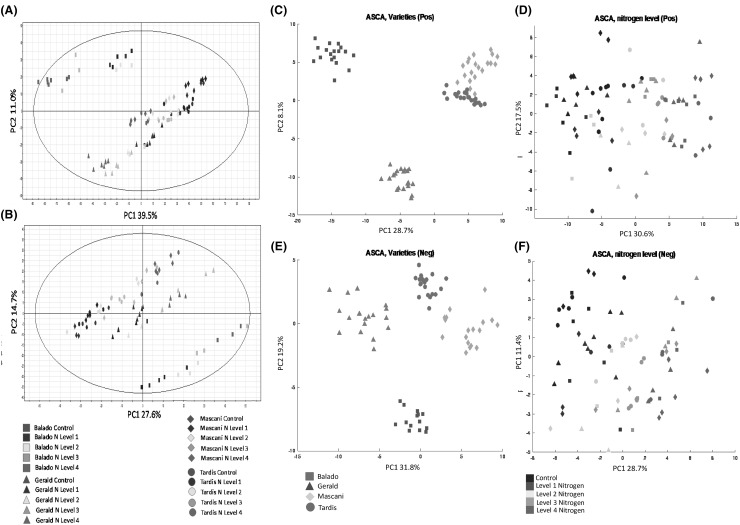
LC-MS Chemometric analyses. Conventional PCA (PC1 × PC2) scores plots: **a** ESI positive, **b** ESI negative. ANOVA-simultaneous component analysis (ASCA): ASCA scores plots **c** ESI positive oat variety, **d** ESI positive nitrogen level, **e** ESI negative oat variety, **f** ESI negative nitrogen level. Control basal nitrogen (58 kg N ha^−1^). Level 1: 50 kg N ha^−1^ supplement (108 kg N ha^−1^). Level 2: 100 kg N ha^−1^ supplement (158 kg N ha^−1^). Level 3: 150 kg N ha^−1^ supplement (208 kg N ha^−1^). Level 4: 200 kg N ha^−1^ supplement (258 kg N ha^−1^). Replication *n*3

Within the first set of PCA models, the QA samples were centrally clustered (X0, Y0) within the scores plots for both the ESI positive and negative mode datasets (data not shown), indicating that the QA samples represented an averaged value of all other sample groups and also that the data processing, filtering, missing value replacement and normalisation, had in no way skewed the ESI positive and negative mode datasets. Within the second set of PCA models where QA samples had been excluded, for the ESI positive mode dataset PC1 x PC2 model (Fig. 2a) the samples revealed a distribution from the positive to negative axis of PC1 associated with the trend of increased nitrogen application, for the ESI negative mode dataset PC1 × PC2 model (Fig. 2b) the converse was observed, where samples revealed a distribution from the negative to positive axis of PC1 associated with increased nitrogen application. Both the ESI positive (Fig. 2a) and negative (Fig. 2b) mode datasets PC1 × PC2 score plots indicated that the Tardis and Mascani oat varieties showed a very closely related metabolic phenotype with both near co-clustering, whereas the Gerald and Balado varieties were much more metabolically distinct and clearly differentiated within the PC2 axis, from each other, as well as from Tardis and Mascani.

Following PCA, ASCA was applied as a method of variable selection rather than purely just data visualisation (Jansen et al. 2005a, b). The constructed ASCA model was designed to test two factors, (i) oat variety (Fig. 2c, e) and (ii) nitrogen level (Fig. 2d, f). The ASCA based results were finally visualised as PCA scores plots, the first factor, oat variety is visualised in the PC1 x PC2 scores plots provided in Fig. 2c (ESI positive) and 2e (ESI negative). Both the ESI positive and negative mode datasets showed similar clustering to that observed in PCA-X for oat variety, with Tardis and Mascani revealing a very closely related metabolic phenotype, whereas Gerald and Balado were much more metabolically distinct and clearly differentiated. This is perhaps not surprising as although Tardis and Mascani are phenotypically distinct for many traits (Table 1), they share a significant level of pedigree in common (Pedigree of Oat Lines (POOL), https://triticeaetoolbox.org/POOL/index_db.php). The second factor, nitrogen level, is visualised in the PC1 x PC2 scores plots provided in Fig. 2d (ESI positive) and 2f (ESI negative), for both ionisation modes, a clear distribution of samples from low to high nitrogen levels were observed from the negative to positive side of the PC1 axis. In addition to ASCA, PLS-S was also employed as a supervised machine learning model (Xu et al. 2016). PLS-S employed a structured response matrix ***Y*** which was coded according to the experimental design and was able to investigate all the studied factors (oat variety and nitrogen level) simultaneously. Recursive feature elimination (RFE; Guyon and Elisseef 2003) was applied with the PLS-S to identify the metabolites that changed significantly in response to increasing nitrogen level regardless of the oat variety in terms of ranks (lower the rank number, higher the importance). The PLS-S models when applied to the test sets with all variables, i.e. no variable selection, generated by bootstrapping (Efron and Tibshirani 1994), revealed 100% correct classification of oats varieties on all the test sets and the regression towards nitrogen level resulted in averaged *Q*^2^ values of 0.9224 and 0.9326 for ESI positive and ESI negative modes, respectively. Given the low levels of sample replication (*n* = 3), the PLS-S model has extremely powerful predictive power.

In addition to the three methods of multivariate statistical analysis, a non-parametric univariate significance test based upon two-way ANOVA (i.e. the Friedman test) was performed, a 5% FDR correction based upon the Benjamini-Hochberg procedure was applied. On the basis of the positive mode dataset, 1584/2012 RT-*m*/*z* pairs were significant at *p* = 0.01 with respect to oat variety, and 1037/2012 with respect to nitrogen application level. Based on the negative mode dataset, 713/924 RT-*m*/*z* pairs were significant at *p* = 0.01 with respect to oat variety, and 413/924 with respect to nitrogen application level. Univariate comparisons were focused on oat samples of each individual variety separately, to define metabolite features that responded in a linear fashion with respect to increasing nitrogen application level. In order to focus primarily on changes in metabolite abundance associated with nitrogen supplementation (rather than inter-varietal differences), the following procedure was followed for each the ESI positive and negative mode datasets individually: features that were the most influential within the PCA (Fig. 2a, b) and the nitrogen level ASCA-PCA (Fig. 2d, f) model loadings were taken forward, the top ranked PLS loadings (99.9% significance cut off) were taken forward, finally the top 100 significant results from the Friedman test were taken forward. The lists of significant features were compiled and reduced where the same feature had been carried forward from multiple statistical tests, or where multiple RT-*m*/*z* pairs relating to a single metabolite feature were still present, thus providing a list of significant features warranting further investigation for each the positive (Table S5) and negative (Table S6) mode ESI datasets. The list of significant features was finally organised according to metabolite class and response to nitrogen supplementation so that the biological and agronomic significance of the results could be thoroughly evaluated.

### Nitrogen supplementation in oats principally affects primary metabolism

**Fig. 3:**
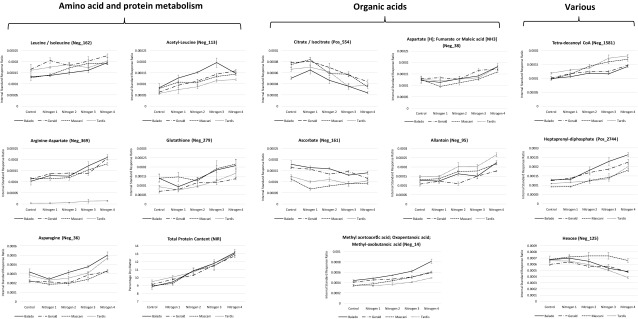
Effects of nitrogen supplementation upon amino acid metabolism and protein synthesis, the TCA cycle and organic acid levels. Control basal nitrogen (58 kg N ha^−1^). Level 1 50 kg N ha^−1^ supplement (108 kg N ha^−1^). Level 2 100 kg N ha^−1^ supplement (158 kg N ha^−1^). Level 3 150 kg N ha^−1^ supplement (208 kg N ha^−1^). Level 4 200 kg N ha^−1^ supplement (258 kg N ha^−1^). LC-MS data is expressed as the internal standard peak ratio, total protein data is expressed as a percentage of dry mass. Replication *n*3. Error bars represent the standard error

The first major impact of increasing nitrogen supplementation on the metabolism of winter oats was the increased content of amino acids within the grain. The observed elevation of amino acids within the grain is likely the result of their import from green tissues, although the elevated nitrogen level may also directly impact upon the synthesis of amino acids within the grain itself. Further studies requiring the measurement of the co-ordinated metabolism between the green plant tissues and maturing oat grain are required for confirmation of which plant tissue(s) show elevated synthesis of amino acids. Of course, the limitation of the current study is that it can only show the impact of increased nitrogen application upon the processed oat grain since the green tissues were not measured. Multiple amino acids that C18 reversed phase chromatography has the ability to retain, were observed to increase linearly as nitrogen availability was increased, inclusive of arginine, asparagine, tryptophan, phenylalanine, leucine and/or isoleucine, as well as modified amino acids inclusive of arginine-aspartate and acetyl-leucine (Fig. 3; Table S3). It is expected that a large number of the amino acid complement would respond in a similar fashion, although many in the case of the rapid UHPLC-MS screening approach applied within this study, were likely lost within the void, or were irreproducible due to elution within the polar front, thus justifying the future application of GC-MS analysis of MOX-TMS derivatives and/or targeted amino acid quantification via HPLC-UV/Vis approaches. For example, it has been previously shown in the leaf tissues of wheat by applying GC-MS analysis of MOX-TMS derivatives that additionally alanine, threonine and tyrosine were elevated when wheat plants were grown in the presence of nitrate in contrast to growth in the absence of nitrate (Allwood et al. 2015). Further to the initial impact on amino acid metabolism within the oat grain, nitrogen elevation in this study was also seen to lead to increased total protein levels downstream, as measured via NIR (Near Infrared) spectroscopy (Tables 1 and S2; Fig. 3), as well as for a number of UHPLC-MS detected tripeptides such as glutathione (Fig. 3).

Nitrate assimilation and amino acid biosynthesis requires NAD(P)H and/or reduced ferredoxin to serve as a supply of reductant, as well as elevated levels of ATP and organic acids to serve as energy and carbon skeleton sources (Glass 2003; Stitt et al. 2002). Although reductant and ATP can be directly assimilated via photosynthesis, mitochondrial respiration may also be a source under specific conditions (Nunes-Nesi et al. 2010). In this case, carbohydrates are converted into organic acids via respiration (glycolysis, the TCA cycle and the oxidative pentose phosphate pathway) to provide an alternative source of carbon skeletons. In which case, nitrate assimilation competes directly with the Calvin cycle for reductant and ATP, thus reducing the total rate of carbon assimilation (Canvin and Atkins 1974), whilst increasing the proportion of assimilated carbon that is incorporated into organic acids and amino acids (Stitt et al. 2002). Interestingly within this study, a number of TCA intermediates that feature in the initial steps of the cycle, namely citrate and isocitrate (Fig. 3), were seen to decrease under conditions of high levels of nitrogen supplementation, whereas an intermediate at the end of the cycle, namely malate, was observed to increase (Tables S4 and S6). These results within the oat grain may suggest that the TCA cycle is potentially being upregulated within green tissues to increase the levels of available ATP and reductant to in turn increase nitrogen assimilation, thus resulting in the early intermediates within the cycle becoming depleted whilst malate formation increased. Interestingly, ascorbate levels within the oat grain were also reduced under conditions of elevated nitrogen availability (Fig. 3), potentially reflecting its role serving as a reductant in order to assist nitrogen assimilation. An unknown hexose sugar and a sugar-alcohol (putatively assigned as arabinitol and/or arabitol and/or ribitol and/or xylitol) were also observed to deplete within the oat grain as nitrogen application increased, potentially highlighting their breakdown in mitochondrial respiration to organic acids thus elevating the availability of carbon skeletons for amino acid metabolism. Measurements of organic acids and carbohydrates, ATP and its intermediates, as well sources of reductant, are required within the developing green tissues as well as the developing non-processed oat grain, to investigate this hypothesis further and to confirm the tissue responsible for their metabolism compared to tissues that likely act as sites of import.

A number of organic acids not associated with the TCA cycle as well as low-MW phenolics were also seen to be elevated under conditions of increased nitrogen availability within the oat grain (Fig. 3; Tables S5 and S6), perhaps indicating their upregulation within green tissues in an effort by the oat plant to increase the availability of carbon skeletons that could be directed into amino acid and downstream protein synthesis. These results indicate the intrinsic links between the carbon and nitrogen assimilation pathways and their requirement for coordination to prevent severe imbalance. Future studies should also consider the importance of potassium to nitrogen balance with respect to the positive impacts that potassium can have on nitrogen update and metabolism in oats (Ahanger et al. 2015). The results also justify the future application of GC-MS analysis of MOX-TMS derivatives, measurement of total carbohydrates and potential application of ZicP-HILIC-MS, for the more precise measurement of TCA intermediates, organic acids and saccharides, within both developing green and grain tissues, to allow a more detailed assessment of the coordination between carbon and nitrogen assimilation pathways, as well as the impact of potassium, within the entire oat plant.

### Nitrogen supplementation in oats leads to elevated levels of nitrogen containing phospholipids but reduced total oil content within the grain

**Fig. 4:**
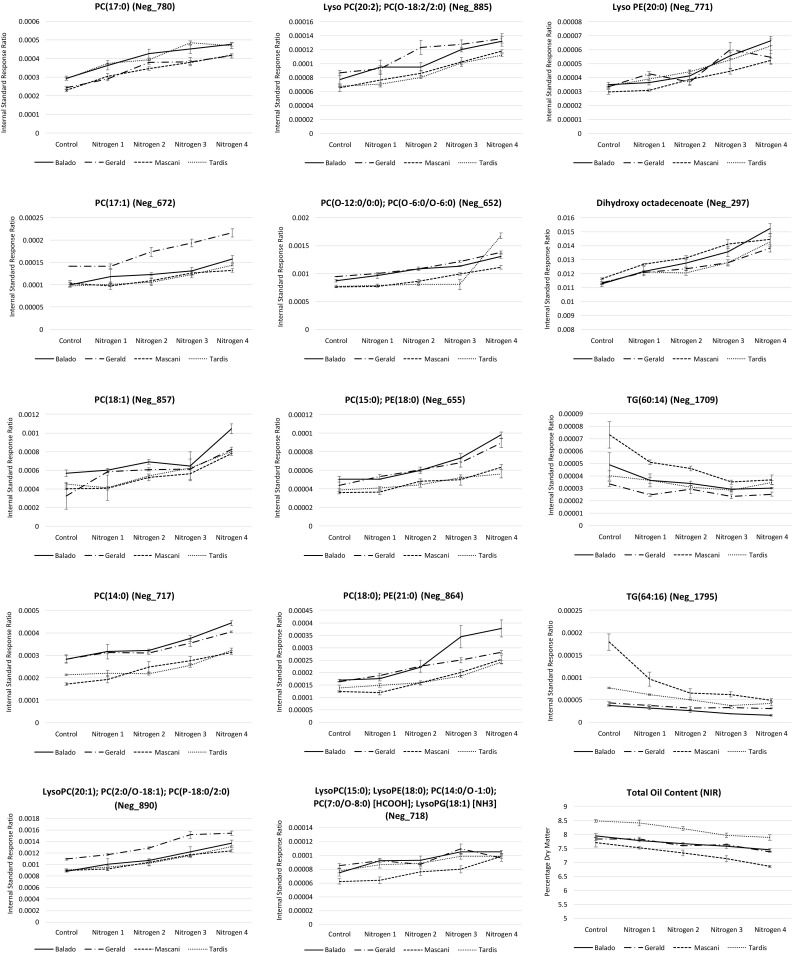
Effects of nitrogen supplementation upon lipid metabolism. Control basal nitrogen (58 kg N ha^−1^). Level 1 50 kg N ha^−1^ supplement (108 kg N ha^−1^). Level 2 100 kg N ha^−1^ supplement (158 kg N ha^−1^). Level 3 150 kg N ha^−1^ supplement (208 kg N ha^−1^). Level 4 200 kg N ha^−1^ supplement (258 kg N ha^−1^). LC-MS data is expressed as the internal standard peak ratio, total protein data is expressed as a percentage of dry mass. Replication *n*3. Error bars represent the standard error

The second major impact of increasing nitrogen supplementation on the metabolism of winter oats within this study was the increased levels of nitrogen containing phospholipids detected within the oat grain. High numbers of both phosphatidylcholines (PCs) and phosphatidylethanolamines (PEs) (Fig. 4) were observed to increase under high levels of nitrogen supplementation. The predominance of PCs and PEs may quite simply be due to the fact that they are purportedly the most represented phospholipid species within the extraplastidic membranes of plants (Nakamura 2017), however the fact that the majority of the increased phospholipids possessed nitrogen containing headgroups is unlikely to be merely coincidental. A number of free fatty acyls were also observed to increase in abundance as the nitrogen supplementation level was increased, however a range of di- and tri-acylglycerols (DGs and TGs), in the case of certain oat varieties (especially Mascani), revealed decreased abundance (Fig. 4). It is tempting to hypothesise that the increased nitrogen level which in turn increases the synthesis of nitrogen containing phospholipids, is placing high demands upon the availability of free fatty acyls, which in turn results in the breakdown of DGs and TGs in order to liberate further fatty acyls.

Given the observation that at the phospholipid species level that nitrogen supplementation increases their abundance, it would be easy to assume that the same would be true of the grains total oil content. However, NIR analysis indicated that at the highest levels of nitrogen supplementation, the oil content was significantly decreased (Tables 1 and S2; Fig. 4). Phospholipids do not alone represent the majority of lipid species within plants and it is perhaps the case that whilst nitrogen supplementation increases the synthesis of nitrogen containing phospholipids, that the demands that their synthesis makes on alternative lipid species such as TGs and DGs to provide fatty acyl constituents, actually results in the slight overall decrease of total oil content. Decreased oil content may also reflect the increased energy demands placed upon the plant in order to assimilate the high levels of available nitrogen for production of amino acids and nitrogen containing phospholipids. To gain a fuller understanding of the impacts of nitrogen supplementation on oat metabolism, it will be key to perform experiments under more controlled conditions than provided within a field study scenario and to perform lipid, amino acid, organic acid and carbohydrate analyses upon both the developing green tissues as well as the developing and mature oat grains, and the final processed oat product.

### Nitrogen supplementation upregulates caffeic and sinapic acid metabolism, but has negative impacts on the levels of health beneficial avenanthramides

**Fig. 5:**
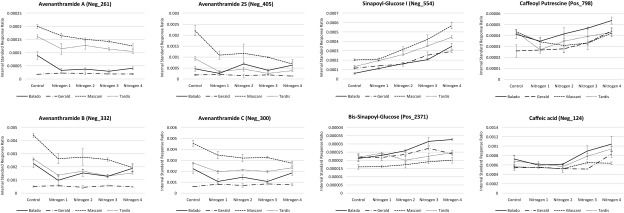
Effects of nitrogen supplementation upon phenolics metabolism. Control basal nitrogen (58 kg N ha^−1^). Level 1 50 kg N ha^−1^ supplement (108 kg N ha^−1^). Level 2 100 kg N ha^−1^ supplement (158 kg N ha^−1^). Level 3 150 kg N ha^−1^ supplement (208 kg N ha^−1^). Level 4 200 kg N ha^−1^ supplement (258 kg N ha^−1^). LC-MS data is expressed as the internal standard peak ratio, total protein data is expressed as a percentage of dry mass. Replication *n*3. Error bars represent the standard error

Elevated nitrogen application was also seen to impact upon areas of secondary metabolism with changes in a number of phenolic compounds being deemed statistically significant. The phenolic compounds that were up-regulated in response to increased nitrogen supplementation included caffeic acid, caffeoyl putrescine and a number of sinapoyl glucose isomers (Fig. 5). Given the antioxidant, antimicrobial, anticancer and antidiabetic activities reported for caffeic acid, sinapic acid and its sinapoyl glucose derivatives (Gülçin 2006; Nićiforović and Abramovič 2014; Eid et al. 2017), this result could potentially be regarded as being positive with respect to oat grain derived health benefits. However, coincidently, a number of health beneficial phenolics, namely the avenanthramides, were seen to be reduced in abundance, in a number of the oat lines, as nitrogen supplementation increased (Fig. 5). This is potentially a result of the elevation observed in primary metabolites effectively limiting precursor availability for the metabolism of the avenanthramides which purportedly takes place in both the green tissues as well as the oat grain (Wise 2017). Avenanthramide production is also thought to be associated with the exposure level to Crown Rust fungus (Wise 2017) and therefore their reduction under increased nitrogen application levels may indicate that the oat plants are subjected to lower burdens of Crown Rust under these conditions. Interestingly, the Balado and Gerald oat varieties that show lower abundance of the avenanthramides were less affected as the nitrogen supplementation level was increased, however the varieties Macani and Tardis, which always showed closer metabolic relativity (Fig. 2), revealed a much greater reduction in avenanthramide abundance as the level of nitrogen supplementation was increased (Fig. 5). As for the other areas of metabolism that were affected by nitrogen supplementation, it would be fascinating to perform more controlled experimentation with sampling of green tissues, as well as oat grains, throughout the development of the plant and grain, applying a large range of metabolomics and transcript expression technologies, to reveal greater insight of carbon and nitrogen metabolism balance, as well as resource partitioning into lipid and secondary metabolism.

## Concluding remarks

The UHPLC-PDA-MS approach developed within this study has provided a rapid, sub 15 min per sample, metabolite profiling method that is repeatable and appropriate for the screening of large numbers of cereal derived grain samples. The method has been shown to be capable of capturing a wide range of compounds in oat, inclusive of primary metabolites such as the amino acids, organic acids, vitamins and lipids, as well as a number of key secondary metabolites, including the health beneficial phenolics such as the avenanthramides, caffeic acid, sinapic acid and its derivatives. A number of interesting insights have been gained from the study, from the more predictable changes in primary metabolism such as those observed to impact upon amino and organic acids, as well as the TCA cycle, through to the remodulation of lipid metabolism, being directed towards the increased production of nitrogen containing phospholipids. A number of both positive (increased levels of caffeic acid, sinapic acid and derivatives) and negative impacts (reduced levels of avenanthramides) on the metabolism of phenolic compounds that have influence upon the nutritional value of oats were also observed. This study has revealed that in addition to the significant enhancement of grain yield and kernel content observed with increased nitrogen application, that there are a number of metabolic consequences, that vary between oat variety, for a wide range of metabolites that may impact on the nutritional quality of the grain.

Although the UHPLC-PDA-MS method has broad applicability as a rapid screening method or a rapid metabolite profiling method that provides greater resolution than the simple application of Direct or Flow Infusion (DI/FI) accurate mass MS, it still must be considered that much greater confidence in metabolite identification, as well as quantitative precision, will be gained by the application of higher resolution chromatography methods in conjunction with accurate mass MS and MS^*n*^ approaches. It is proposed that in follow up studies that oats will be grown in multiple locations as well as multiple growth seasons, effectively providing a cross validation for the results obtained within this preliminary study. The oats will be subjected to methanol-chloroform-water extraction procedures, thus providing a polar phase extract that will be subjected to MOX-TMS derivatisation and GC-MS analysis, as well as higher resolution C18 reverse phase LC-MS of phenolics and ZicP HILIC-MS of sugars and organic acids, and a non-polar phase extract that will be subjected to higher resolution C18 reverse phase LC-MS of lipids. Since amino acid metabolism is such a key area of importance in the study of nitrogen metabolism, it will also be of importance to subject samples to targeted methods for their precise quantification in the future.

In the future, it will also be fascinating to perform more controlled experimentation with sampling of green tissues, as well as oat grains, throughout the development of the plant and grain, to reveal greater insight of carbon and nitrogen metabolism balance, the effect of enhancing the application rates of other nutrients such as phosphate, and tracking resource partitioning into lipid and secondary metabolism. Controlled studies across multiple tissue types will be key to our future understanding of nitrogen metabolism in cereals, such studies are currently extremely limited in oats and are even restricted for the more commonly studied cereals such as wheat. Controlled studies across multiple cereals i.e. both wheat and oats, will be of benefit though since common results between both cereals can be drawn upon further, especially in the context of the significance of previous data collected only in wheat. Such controlled studies in oats could also finally tie down the relationship between soil nitrogen addition, variety and grain β-glucan level, which remains elusive or according to the existing reports, contradictory.

## Electronic supplementary material

Below is the link to the electronic supplementary material.

Supplementary material 1 (DOCX 16 KB)

Supplementary material 2 (XLSX 23 KB)

Supplementary material 3 (XLSX 2807 KB)

Supplementary material 4 (XLSX 1321 KB)

Supplementary material 5 (XLSX 87 KB)

Supplementary material 6 (XLSX 211 KB)

## Data Availability

Metabolomics data have been deposited to the EMBL-EBI MetaboLights database (DOI: 10.1093/nar/gks1004. PubMed PMID: 23109552) with the identifier MTBLS804. The complete dataset can be accessed here https://www.ebi.ac.uk/metabolights/MTBLS804.
