# Supplementary material for: Rapid UHPLC-MS metabolite profiling and phenotypic assays reveal genotypic impacts of nitrogen supplementation in oats
Source: Metabolomics. 2019 Mar 12;15(3):42. doi: 10.1007/s11306-019-1501-x (PMC6476850; doi:10.1007/s11306-019-1501-x)
Supplement: Supplementary file 1 — Supplementary material 1 (DOCX 16 KB) [file 11306_2019_1501_MOESM1_ESM.docx]

| N LEVEL | *4^th^ April*  *(kg N/ha)* | *17^th^ April*  *kg N/ha* | *2^nd^ May*  *(kg N/ha)* | *Total Nitrogen applied*  *(kg N/ha)* |
| --- | --- | --- | --- | --- |
| Control | *0* | *0* | *0* | *0* |
| Level 1 | *0* | *50* | *0* | *50* |
| Level 2 | *50* | *50* | *0* | *100* |
| Level 3 | *50* | *50* | *50* | *150* |
| Level 4 | *50* | *75* | *75* | *200* |

Rapid UHPLC-MS metabolite profiling and phenotypic assays reveal impacts on the levels of amino acids, TCA cycle intermediates, lipids and phenolic metabolites, in response to nitrogen supplementation in winter oats

*Metabolomics*

J. William Allwood*, Yun Xu, Pilar Martinez-Martin, Raphaёlle Palau, Alexander Cowan, Royston Goodacre, Athole Marshall, Derek Stewart, Catherine Howarth

*Environmental and Biochemical Sciences, James Hutton Institute, Invergowrie, Dundee, DD2 5DA

Email: Will.Allwood@hutton.ac.uk

Table S1: Nitrogen fertiliser applied (kg/ha), dates and doses of application
